# Improving the quality of life of parents of patients with congenital abnormalities using psychoeducational interventions: a systematic review

**DOI:** 10.1007/s11136-023-03452-8

**Published:** 2023-06-17

**Authors:** Marisa Garcia Rodrigues, José Daniel Rodrigues, Matilde Monteiro Soares, Luís Filipe Azevedo, Pedro Pereira Rodrigues, José Carlos Areias, Maria Emília Areias

**Affiliations:** 1 Centro Hospitalar Universitário São João (CHUSJ), Porto, Portugal; 2 Centro de Investigação em Tecnologias e Serviços de Saúde (CINTESIS), Faculdade de Medicina da Universidade do Porto (FMUP), Porto, Portugal; 3 Departamento Medicina da Comunidade, Informação e Decisão em Saúde (MEDCIDS), Faculdade de Medicina da Universidade do Porto (FMUP), Porto, Portugal; 4 Unidade de Investigação Cardiovascular da Faculdade de Medicina da Universidade do Porto (UnIC), Porto, Portugal; 5 Faculdade de Medicina da Universidade do Porto (FMUP), Porto, Portugal; 6 Instituto Universitário de Ciências da Saúde (IUCS), Gandra, Portugal

**Keywords:** Psychoeducational interventions, Quality of life, Parents, Children, Congenital abnormalities, Congenital heart defects

## Abstract

**Purpose:**

To identify psychoeducational interventions that target parents of children with congenital abnormalities (CA) and evaluate their impact on quality of life (QoL).

**Methods:**

The search was conducted in six electronic databases*,* complemented by references of the studies found, studies of evidence synthesis, a manual search of relevant scientific meetings’ abstracts and contact with experts. We included primary studies on parents of children with CA that studied psychoeducational interventions *versus* standard care. We assessed the risk of bias using *Cochrane Collaboration’s tool.*

**Results:**

We included six studies focusing on congenital heart defects (CHD). They described four different psychoeducational strategies. In four studies, statistically significant differences were found. For clinical practice, we considered three interventions as more feasible: the *Educational program for mothers*, with a group format of four sessions weekly; *CHIP-Family intervention,* which includes a parental group workshop followed by an individual follow-up booster session; and *WeChat educational health program* with an online format.

**Conclusions:**

This review is the first that assesses the impact of psychoeducational interventions targeted at parents of children with CA on their QoL. The best approach to intervention is multiple group sessions. Two essential strategies were to give support material, enabling parents to review, and the possibility of an online program application, increasing accessibility. However, because all included studies focus on CHD, generalizations should be made carefully. These findings are crucial to guide future research to promote and improve comprehensive and structured support for families and integrate them into daily practice.

**Supplementary Information:**

The online version contains supplementary material available at 10.1007/s11136-023-03452-8.

## Introduction

The World Health Organization (WHO) defines congenital abnormalities (CA) as structural or functional abnormalities that occur in the prenatal period [1]. Their diagnosis can be made in the prenatal period, at birth or only later [1]. The WHO estimates that almost 300,000 newborns die every year in the first 28 days of life due to CA [1]. CA also contribute to long-term disability, significantly impacting individuals and their families [1].

Congenital heart defects (CHD) represent one third of all CA, making them the most common [2]. Their prevalence varies between 6.9 per 1000 live births in Europe and 9.3 in Asia [3]. Over one third of infants undergo surgery in the first months of life, and more than 60% of parents are affected by stress or depression [4].

The diagnosis of a child’s CA challenges the parents’ expectations of a healthy baby and defines the dynamics and functioning of the family [5]. Parents face additional demands on the level of care they must provide, high levels of psychological distress, economic consequences, and an impact on their quality of life (QoL) [5–7]. These circumstances influence the entire family and impose the restructuration of their responsibilities and purposes [6, 8]. In the absence of an adequate response, parents present high levels of psychological symptoms, namely anxiety and depression, and a severe impact on their QoL [8]. Numerous reports account for a lower QoL in parents of children with a CA, which may negatively impact the children [5–9]. The literature also suggests that family adaptation, coping strategies, and general functioning significantly influence children’s adaptation to the condition and treatment, affecting the therapeutic inclusively [8]. Social support is essential to protect against stress, promote psychosocial integration, and improve QoL [10]. Nevertheless, until now, the research focus has been on children’s QoL, and parents have been forgotten.

Family-oriented psychosocial interventions must integrate the rehabilitation paradigm. Empowering parents, physically and mentally, improves their capability and affects chronically ill children’s well-being and development [8, 9, 11]. Lazarus and Folkman’s (1984) theory of stress and coping distinguishes two primary coping forms: problem focused and emotion focused [12]. Fonseca et al*.* (2012) reported that parents searching for information after diagnosing a CA is an essential coping mechanism [6]. Considering Griffin’s work (2002), mothers with children with CHD need more information which increases their confidence and self-esteem [10]. As defined by Lukens (2015), psychoeducation “is a flexible strengths-based approach to care that incorporates both educational and therapeutic techniques and can be adapted to serve those with various medical, psychiatric, and other life challenges” [13]. The educational element delivers critical knowledge and treatment approaches regarding sickness or life challenges [13]. It can be offered individually or in groups [13]. The group setting allows the exchange of stories, knowledge, and collective assistance, which can enrich the involvement of participants [13]. The psychotherapeutic element provides to participants security, structure, comment, and a moment to integrate the data that may be different and intriguing, which can lead to complex emotions [13].

One cannot find an ideal definition of psychoeducation but must agree to incorporate the following principles: (1) a discussion between professionals and participants; (2) an organization that makes it simple and easy for participants to attend without significant constraints and to get the necessary information, either general information about stress and illness-specific information; (3) programmed time for handling material and feelings; (4) strategies to improve functioning and QoL and to lessen burden and stigma; (5) watchful consideration to the adjustment of information and timing, and process grounded on social setting [13]. Different studies report a relationship between parents’ QoL and psychopathological symptoms with the clinical state of their ill children and vice versa. These data support the implementation of interventions directed to parents’ mental health and QoL, consequently helping in the recovery and well-being of these patients [6, 7, 14–17]. This knowledge encourages creating psychoeducation groups and a family approach as an essential focus in treating ill children [8].

The primary aim of this systematic review was to assess the impact of psychoeducational interventions targeted at parents of children with CA on their QoL.

This study is part of a research project aiming to evaluate the impact of a psychoeducational intervention on the QoL of parents of patients with CHD (ClinicalTrials.gov Identifier: NCT03724006).

## Methods

### Search

The search was conducted in the bibliographic databases: MEDLINE (Pubmed), SCOPUS, Web of Science, Cochrane Central Register of Controlled Trials (CENTRAL), and PsycINFO by one investigator (JDR). To identify ongoing clinical trials, he also searched *ClinicalTrials.gov*. The development of the query search was an iterative process in which controlled vocabulary, free text, synonyms, and related terms were used, connected by Boolean operators. We used four main concepts: parents, CA, QoL, and education. The query search and respective adaptations to different databases are presented in Attachment 1 (electronic supplementary material). The last search was performed on 14th May 2022. No restriction on language or date of publication was applied.

The search was complemented by references to the studies found and studies of evidence synthesis. In addition, we conducted a manual search of abstracts of relevant congresses and scientific meetings held in the last eight years (Attachment 1—electronic supplementary material).). Lastly, experts in this area of knowledge were contacted, as well as the authors of the articles found, in case additional clarifications were required.

### Study selection

The inclusion criteria considered were (1) primary studies on parents of children with CA; (2) assessing psychoeducational interventions *versus* standard care; (3) defining as a primary outcome the QoL of parents of children with CA; and (4) using quantitative comparative observational or experimental designs.

Other studies like non-comparative observational studies, qualitative studies, letters, systematic reviews, narrative reviews, and case reports were excluded.

In the screening phase, we analyzed the articles’ titles and abstracts. During the inclusion phase, all papers were selected by reading their integral text. Both steps were carried out by two reviewers (MGR and JDR) blindly and independently. The reason for exclusion was recorded using an eligibility checklist—Attachment 2 (electronic supplementary material). A third reviewer solved the disagreements (MMS). The reproducibility of the selection process was evaluated using the proportion agreement.

### Data extraction

Data were collected through a specific form that was subjected to a pilot study. We extracted the following characteristics: general characteristics of the study (aim, study design, time frame, setting, sample size, and sampling); sociodemographic characteristics of the sample (sex, age, marital and socioeconomic status); children’s features (age, type of CA and their severity); description of the psychoeducational intervention and respective duration as well of the standard care; methods of assessment; and the results obtained in QoL. Whenever possible, QoL scores and respective estimations of precision were extracted (when necessary, the authors were contacted). As in the selection phase, the extraction was carried out by two reviewers (MGR and JDR) blindly and independently, and a third reviewer (MMS) was used to solve disagreements.

#### Risk of bias (quality) assessment

We evaluated the risk of bias/quality of the studies using *Cochrane Collaboration’s tool for assessing risk of bias* [18]. Two reviewers (MGR and JDR) did the quality evaluation blindly and independently. A third reviewer solved the disagreements (MMS).

### Strategy for data synthesis

The qualitative synthesis aimed to identify the psychoeducational interventions described in the literature and assess their effectiveness in improving parental QoL. This analysis was organized by study design, CA, psychoeducational intervention, and QoL assessment method.

The quantitative data extracted from the primary studies were analyzed to decide whether it was suitable to perform quantitative synthesis through a meta-analysis. Heterogeneity was assessed using the Cochran Q test (significance level of 0.05), supplemented by the *I*^2^ statistic. We used the Random Effects Model. When it did not make sense to compute and present a meta-analytic measure of the effect size of psychoeducational interventions (*I*^2^ > 40–50%), the exercise of an explanatory attempt of variability was carried out. To compute the effect size, we used Cohen’s *d*. The interpretation of effect size values was made considering the cut-offs presented by Cohen in 1988 [19]. Values of 0.20, 0.50, and 0.80 for *Cohen’s d* are commonly considered to be indicative of small, medium, and large effects [19].

EndNote® software was used for reference management. Covidence® software was used in the selection phase and data extraction. With the help of Open Meta-Analyst® software, quantitative data were analyzed.

This study followed the orientations included in the *Cochrane Handbook for Systematic Reviews of Interventions* and *PRISMA Statement* [18, 20].

The protocol of this review was registered in PROSPERO, an international prospective register of systematic reviews, with the number *PROSPERO 2017 CRD42017079534.*

Available from: https://www.crd.york.ac.uk/prospero/display_record.php?ID=CRD42017079534

## Results

### Search

**Fig. 1:**
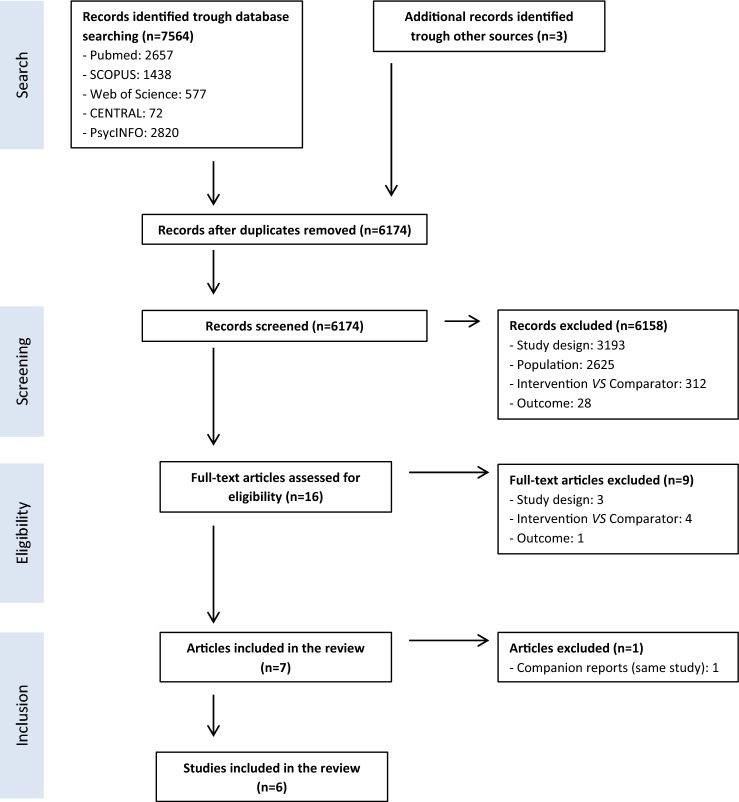
Flow chart illustrating study selection process following PRISMA (Preferred Reporting Items for Systematic Reviews and Meta-Analyses) Statement

Figure 1 presents the flow chart of the selection process with the mention of the reasons for exclusion. We found 7567 records, 7564 from the bibliographic databases, and three from other sources. From these, 1393 were identified as duplicates and removed. We did the screening of 6174 records and excluded 6158 of them. The agreement proportion of the screening phase was 0.99.

In the inclusion phase, we reviewed the full text of 16 records and excluded 9, with a proportion of agreement of 1. So, we identified seven reports that met the inclusion criteria [7–9, 21–26]. Because our unit of analysis is the study and not the papers, we included six studies [7, 8, 21, 23–26] (one paper was a companion report of the same research) [21].

### Description of studies

In Table 1, we present a summary of the included studies. A detailed characterization of the research question using PICOS structure (P—Population; I—Intervention; C—Control; O—Outcome; and S—Study type) of included studies is shown in Table 2.

**Table 1:** Description of included studies by country, study design, sample size, number of parents, type of congenital abnormalities, range of children’s age, psychoeducational intervention, and parental QoL assessment tool

Study	Country	Study design	Sample size	No. of parents, (% mothers)	Congenital Abnormalities	Children’s age range	Psychoeducational intervention	Parents’ QoL assessment tool
Experimental								
Edraki et al. [7]	Iran	Randomized controlled trial	56	56 (100)	Congenital heart disease	< 1 year	Educational program for mothers	SF-36
Hancock et al. [21]	USA	Randomized controlled trial	38	38 (100)	Congenital heart disease	*Neonates*	Early structured pediatric palliative care intervention	PedsQL FIM
van der Mheen et al. [22]^a^	Netherlands	Randomized controlled trial – study protocol	Target: 90	90 (NR)	Congenital heart disease	4 to 7 year	CHIP-Family	SF-36
van der Mheen et al. [23]	Netherlands	Randomized controlled trial	154	154 (52.6)	Congenital heart disease	2 to 8 year	CHIP-Family	SF-36
Zhang et al. [24]	China	Randomized controlled trial	70	70(NR)	Congenital heart disease (restrictive ventricular septal defects)	< 1 year	WeChat-assisted pre-operative health education	WHOQOL-Bref
Zhang et al. [25]	China	Randomized controlled trial	168	168(50)	Congenital heart disease	< 1 year	WeChat-assisted post-operative health education	WHOQOL-Bref
Xie et al. [26]	China	Randomized controlled trial	80	80(NR)	Congenital heart disease (unrestrictive ventricular septal defects)	< 6 months	WeChat-assisted post-operative health education	PedsQL FIM

*NR* not reported, *SF-36* SF-36 Health Survey, *PedsQL FIM* Pediatric Quality of Life Inventory Family Impact Module, *WHOQOL-Bref* World Health Organization Quality of Life Bref

^a^Companion reports (same study): van der Mheen et al. (2018) and van der Mheen et al. (2019)

**Table 2:** Characterization of the PICOS research question structure of included studies

References	Population (P)	Intervention (I)	Control (C)	Outcomes (O)	Type of study (S)
Edraki et al. [7]	Mothers who had infants with CHD (Atrial Septal Defect, Ventricular Septal Defect, and Patent Ductus Arteriosus)	Educational program	Routine care without any educational program	Quality of life (SF-36) Self-efficacy (Generalized Self Efficacy scale) Measurement moments: T0—before the intervention; T1—immediately after the intervention; T2—2 months after the intervention	Randomized controlled trial
Hancock et al. [21]	Mothers of neonates with single-ventricle heart disease that were admitted for planned surgery – following birth but before the 1^st^ stage of single-ventricle palliative surgery	Early structured pediatric palliative care	Standard care (no or late palliative care consultation)	Quality of life (PedsQL FIM) Depression (Beck Depression Inventory-II) Anxiety (State-Trait Anxiety Index) Coping (Brief Cope Inventory) Measurement moments: T1—follow-up prenatal visit; T2—neonatal hospital discharge (or 30 days) following the 1^st^ stage palliative surgery	Randomized controlled trial
van der Mheen et al. [22, 23]	Families of children who underwent at least one invasive procedure for CHD (cardiac catheterization and/or open-heart surgery) and are starting or attending kindergarten or first or second year of primary school at the time of first assessment [children, parents and siblings]	CHIP-Family intervention	Care as usual (no psychosocial intervention)	Parental Quality of Life (SF-36) Parental mental health (SCL-90) Disease-specific knowledge and illness perception (Rotterdam Knowledge Questionnaire for Congenital Heart Disease) Parental worry (Penn State Worry Questionnaire: PSWQ) Parenting stress (Nijmeegse Ouderlijke Stres Index verkort: NOSIK and Distress Thermometer-P: DT-P) Family functioning (Family Assessment Device: FAD) Program satisfaction (social validity questionnaire) Measurement moments: T1—baseline (2 weeks before intervention); T2—follow-up (6 months after T1)	Randomized controlled trial
Zhang et al. [24]	Parents of children with restrictive ventricular septal defects who will go under surgical treatment	WeChat-assisted pre-operative health education	A leaflet with the same educational information as that given to the intervention group	Quality of Life (WHOQOL-Bref) Anxiety (Self-Rating Anxiety Scale: SAS) Measurement moment: pre-operative period	Randomized controlled trial
Zhang et al. [25]	Both parents of infants with CHD who underwent surgical treatment	WeChat-assisted post-operative health education	A leaflet with the same educational information as that given to the intervention group	Quality of Life (WHOQOL-Bref) Anxiety (SAS) Depression (Self-Rating Depression Scale: SDS) Measurement moment: 1 month after surgery	Randomized controlled trial
Xie et al. [26]	Parents of children with restrictive ventricular septal defects after undergoing ventricular septal defect repair	WeChat-assisted post-operative health education	Followed up with a traditional method	Quality of life (PedsQL FIM) CHD knowledge (Leuven congenital heart disease knowledge questionnaire: LKQCHD) Measurement moment: 3 months after surgery	Randomized controlled trial

*CHD* Congenital Heart Disease, *SF-36* SF-36 Health Survey, *PedsQL FIM* Pediatric Quality of Life Inventory Family Impact Module, *WHOQOL-Bref* World Health Organization Quality of Life Bref

The included studies were published from 2014 through 2021 and were performed in four countries that correspond to three continents—Europe [22], North America [21], and Asia [7, 24–26]. Considering the type of study, we included six experimental studies (randomized controlled trials—RCT) [7, 21, 23–26]. A total of 566 participants were assessed (ranging from 38 to 168 per study). Four studies included fathers and mothers [8, 23–26], and the other two had considered merely mothers [7, 21]. The included studies focused on the most frequent type of CA, CHD. Children’s age ranged from 0 to 8 years old.

We identified three different QoL assessment tools: SF-36 Health Survey (SF-36) [7, 23], WHOQOL-Bref [24, 25], and PedsQL Family Impact Module (PedsQL FIM) [21, 26]. SF-36 and WHOQOL-Bref are generic measures of individual QoL. In contrast, PedsQL FIM is a generic tool to assess family QoL. It measures the impact of pediatric chronic health conditions on parents’ HRQOL and family functioning.

### Psychoeducational interventions

The included studies described four different interventions, of which description, QoL results, and effect sizes estimation are summarized in Attachment 3 (electronic supplementary material).

Edraki et al*.* (2014) evaluated the effect of an *Educational program on mothers of infants with CHD* [7]. The training was performed in a small group setting (four subjects) through four 90-min sessions over four weeks. The themes presented were information about the disease: its types, causes, symptoms, diagnostic tests, and treatments; its effect on the infant and family; coping methods; taking care of such infants at home; nutrition; preventing infection; vaccination; and medication. Participants received a booklet on the approached themes.

Significant differences were observed between the study and control groups regarding the mean QoL assessed immediately after [PCS 47.6(28.1); MCS 45.33(25.6) vs PCS 46.1(29.2); MCS 26.6(27.2)], and 2 months after the training [PCS 47.2(27.9); MCS 41.4(26.0) vs PCS 45.6(29.9); MCS 26.7(26.0)] with *p* value = 0.001. The study group obtained higher SF-36 scores that correspond to better QoL. The Cohen’s *d* of MCS was 0.71 and 0.57 between the end and 2 months after the intervention. These values correspond to a medium effect size.

Hancock et al*.* (2018) described an *Early structured pediatric palliative care* that consists of an intervention for mothers of neonates with CHD (single-ventricle heart disease) performed during their hospitalization for elective surgery (before the first-stage palliative surgery) [21]. They made an initial palliative care consultation and one to four weekly follow-up appointments. The main topics communicated were parental knowledge regarding diagnosis and life impact; apprehensions concerning the child’s physical manifestations; social care and other life anxiety factors; outlooks and faiths for their child’s medical attention; and worries adjacent to their child’s diagnosis and treatments. Every intervention focused on these topics and emphasized three critical questions: “What is your understanding of your baby’s diagnosis and how it might affect his/her and your family’s lives?”; “What are you and your family hoping for?”; and “What are you most worried about?” [21]. The answers were fundamental to program the subsequent intervention, namely the number and length of the following appointments. There were no statistically significant differences between groups in total PedsQL FIM score [study group 60.0(13.9) vs control group 60.2(20.5)]; family functioning summary [study group 65.4(18.6) vs control group 68.4(23.4)] and parent HRQOL summary [study group 57.2(16.7) vs control group 60.5(24.6)]. The effect size between groups was insignificant (Cohen’s *d* < 0.2). Nevertheless, the results indicated a beneficial difference in the scores of communication and family relationships scales for the PedsQL FIM [21].

The first psychosocial intervention mainly focused on parents and children with CHD—*CHIP-Family intervention*—was performed by van der Mheen et al*.* [23]. Parents attended the first workshop, directed to psychoeducation, problem prevention, general and specific skills, plus an individual follow-up booster session four weeks later. The lunch break was viewed as an opportunity to interact and share experiences. They also received a handbook summary of the subjects covered throughout the meeting and a home assignment directed to problem prevention therapy. About four weeks later, the parents received the individual follow-up booster session. Questions that arose after the first meeting about their ill child or their family members were answered, and the problem prevention home assignment was reviewed. Considering parental QoL, no statistically significant differences between the intervention and control groups were found [23]. We found a small effect size in mothers’ MCS (Cohen’s *d* 0.22) and fathers’ PCS and MCS (0.32 and 0.44, respectively).

Zhang et al*.* [24] studied *WeChat-assisted pre-operative health education*. Zhang et al. [25] and Xie et al*.* [26] investigated *WeChat-assisted post-operative health education*. Both programs included two modules: education and question and answer. The first one focuses on CHD knowledge, pre- or post-operative care, family care, feeding, and complications’ management. Parents could view the module and learn at any convenient time. In the question-and-answers module, one medical staff member of the team was on duty every day and was online in the WeChat group from 18:00 to 22:00 h to address parents’ problems. The medical staff also guided the family members in the WeChat group to communicate, discuss, share experiences, and support each other.

In Zhang et al*.* (2021) research, WHOQOL-Bref results of the two groups had a statistically significant difference in the pre-operative period [study group: Physiological 12.5(1.9); Psychological 14.8(2.8); Social 14.3(2.4) and Environment 13.5(2.2) vs control group: Physiological 9.7(1.3); Psychological 10.2(1.5); Social 10.6(1.2); and Environment 9.9(1.6)] [24]. For all the WHOQOL-Bref domains, Cohen’s *d* was > 0.8, which traduces a large effect size.

In Zhang et al*.* (2021) study, WHOQOL-Bref results from one month after surgery presented statistically significant differences between the two groups [study group: Physiological 15.6(3.1); Psychological 16.2(2.9); Social 16.5(3.0) and Environment 15.8(2.8) vs control group: Physiological 10.8(3.3); Psychological 10.2(3.5); Social 9.7(3.6); Environment 9.9(3.1)] [25]. These results traduce a large effect size (Cohen’s *d* > 0.8) in all domains except in the Physiological one, which is medium (Cohen’s *d* 0.75).

Xie et al*.* (2021) found in their work a statistically significant difference between the groups [study group: Total impact score 70.8(7.1); Family functioning summary 70.6(13.1) vs control group: Total impact score 62.6(6.3); Family functioning summary 63.0(15.7)] with a *p* value of < 0.001 and 0.013, respectively [26]. For the Total impact score, the size effect was 1.22 (large), and the Family functioning summary corresponded to an effect size of 0.53 (medium).

#### Risk of bias (quality) assessment

In Table 3, we present the results of the quality evaluation. Five included studies have at least one parameter classified as *High risk* [8, 21, 23–26], and one study has two parameters classified as *Unclear* [7]*.*

**Table 3:** Risk of bias (quality) assessment of included studies

Cochrane Collaboration’s tool for assessing risk of bias [31]						
Domain	Random sequence generation	Allocation concealment	Blinding of participants and personnel	Blinding of outcome assessment	Incomplete outcome data	Selective reporting
Edraki et al. [7]	Low risk	Low risk	Unclear	Unclear	Low risk	Low risk
Hancock et al. [21]	Low risk	Unclear	High risk	High risk	Low risk	Low risk
van der Mheen et al. [22]	Low risk	Low risk	High risk	Low risk	Low risk	Low risk
van der Mheen et al. [23]^**a**^	Low risk	Unclear	High risk	Low risk	NA	NA
Zhang et al. [24]	Low risk	Unclear	High risk	High risk	Low risk	Low risk
Zhang et al. [25]	Low risk	Low risk	High risk	Unclear	Low risk	Low risk
Xie et al. [26]	Low risk	Unclear	High risk	Unclear	Low risk	Low risk

Companion reports (same study): van der Mheen et al. (2019) and van der Mheen et al*.* (2018)

^a^Study protocol; *NA* not assessed

Hancock et al. (2018), van der Mheen et al. (2019), Zhang et al. (2021), Zhang et al. (2021), and Xie et al. (2021) researches were evaluated as *High risk* for blinding participants and personnel [21, 23–26].

Hancock et al. (2018) and Zhang et al. (2021) investigations were considered *High risk* for blinding outcome assessment [21, 24]. Finally, Edraki et al.’s (2021) investigation risk assessment was classified as *Unclear* for blinding participants and personnel plus outcome assessment [7].

We did not perform quantitative synthesis because the interventions identified were very heterogeneous. In addition, the instruments/tools used to assess the outcome QoL were not comparable, and the small number of studies included did not allow us to do subgroup analysis. However, we presented the QoL results and the effect size estimation in Attachment 3.

## Conclusions

We included six studies and identified four psychoeducational interventions. The interventions described were very heterogeneous, although they all aimed to improve the adaptation to the diagnosis and management of the disease and increase health outcomes for the whole family. They differed in duration, content, target population, and QoL assessment instrument.

Relatively to the duration of the psychoeducational interventions, Edraki et al. (2014) described four weekly 90-min sessions [7]; Hancock et al. (2018) between 2 and 8 consultations (with a median of 3) [21]; van der Mheen et al. (2019) a 6-h group workshop followed by an individual booster session [23]. Zhang et al. (2021), Zhang et al. (2021), and Xie et al. (2021) evaluated a *WeChat health education program* in which parents could watch and learn at any convenient time [24–26]. So, the four interventions were implemented in the ambulatory context and could be divided according to their duration into short [22] *versus* long [7, 21, 24–26]*.*

For clinical practice, we considered three of the described interventions as more feasible: (i) the *Educational program for mothers* [7], with a group format of four sessions weekly; (ii) *CHIP-Family intervention* [23], which includes a parental group workshop followed by an individual follow-up booster session, and (iii) *WeChat educational health program* with two components: educational module and question-and-answer module [24–26].

Nevertheless, we can point out the strong and weak aspects of the three approaches. On one side, the format of several group sessions in the *Educational program for mothers* allows participants to develop a sense of group identity, share worries and doubts, and learn, practice, and ask questions during the intervention. However, the four meetings increase the probability of dropouts, and the group format could not be the best approach for all. On another side, *CHIP-Family intervention* that uses two kinds of techniques, group and individual, has both benefits. The short duration of intervention, one group workshop and an individual follow-up booster session reduce the occurrence of dropouts. However, the group workshop is very long (6 h), which could be exhausting. Being a single group session may be difficult for some parents to share experiences, thoughts and worries with the group. Lastly, in the *WeChat education program*, the parents could complete it at the most convenient time, according to their availability. Additionally, because it is an online program, it avoids the inconvenience of dislocation and allows access to the program to rural populations that live away from hospitals. However, some parents could not feel comfortable sharing their experiences online.

Although the target population of all included studies was parents of children with CHD, the most prevalent form of CA, the spectrum of CHD severity was very wide. On one extreme, we had the work of Edraki et al. (2014), who studied mothers of children with mild CHD; on the opposite extreme, we had Hancock et al. (2018), whose population studied was composed of mothers of children with severe CHD. Because the included studies focused on parents of children with CHD, our conclusions could not be generalized to CA without extreme caution, representing a limitation of the present systematic review.

Another aspect to consider was the range of children’s age, from less than one year to 8 years old.

Moreover, two of the studies included mothers exclusively, whereas the other four included mothers and fathers. The literature described different patterns of adaptation to the diagnosis of a child’s CA by mothers *versus* fathers [4, 27, 28]. Despite the gender differences reported in the adjustment process, assessing the impact of diagnosis and interventions that promote successful adaptation should ideally target both parents [28, 29].

It is essential to reflect on the design of studies to decrease some sources of bias. Although all included studies are RCT, the nature of the intervention studied makes it hard to control some aspects that increase the risk of bias, like blinding.

Three instruments were used to assess QoL outcome: SF-36, PedsQL FIM, and WHOQOL-Bref. This variety makes difficult the presentation of a quantitative analysis of the results. The SF-36 is a generic measure of functional health and well-being, whose results could be reported in two scores, the physical and the mental component summary (PCS and MCS, respectively). The PedsQL FIM measures the impact of pediatric chronic health conditions on parents’ HRQOL and family functioning. It scores parental HRQOL summary and family functioning summary along with the total impact score. WHOQOL-Bref is a generic QoL assessment tool that evaluates physical, psychological, social, and environmental domains.

From a practice perspective, PedsQL FIM has advantages relative to SF-36 and WHOQOL-Bref because it is a tool specially designed to assess parents with chronically ill children.

The reported results about the effectiveness of psychoeducational interventions on parental QoL were not uniform. However, the majority—Edraki et al. (2014), Zhang et al. (2021), Zhang et al. (2021), and Xie et al. (2021)—showed significant differences between the study and the control groups. In contrast, Hancock et al. (2018) and van der Mheen et al. (2019) did not find significant differences. This evidence suggests that multiple group sessions are the best approach to psychoeducational intervention for parents with children with CHD. It is hard to draw the program’s content from these data, limited to a few studies. An important strategy is to give support material on the most critical themes enabling parents to review them.

The literature supported the implementation of interventions that focus on the whole family system [30, 31]. Smith and Grzywacz’s (2014) results were consistent with previous works that corroborate the resilience framework. Through protective factors, such as parents’ sense of control and social support, parents of children with special health care needs can thrive despite additional challenges associated with the parenthood of a child with special health care needs. So, it is essential to implement interventions that increase support for these families.

The present review is the first to assess the impact of psychoeducational interventions targeted at parents of children with CA on their QoL. The results highlight a gap in the content and design of psychoeducational interventions targeted to this population. This finding is essential to direct future efforts to research this subject in order to allow comprehensive and structured support to these vulnerable families based on solid evidence.

### Supplementary Information

Below is the link to the electronic supplementary material.

Supplementary file1 (PDF 2766 kb)

## References

[1] Organization WH. (2020). Congenital anomalies. Retrieved from https://www.who.int/news-room/fact-sheets/detail/congenital-anomalies

[2] Dolk H, Loane M, Garne E (2011). Congenital heart defects in Europe: Prevalence and perinatal mortality, 2000 to 2005. Circulation.

[3] Van Der Linde D, Konings EE, Slager MA, Witsenburg M, Helbing WA, Takkenberg JJ, Roos-Hesselink JW (2011). Birth prevalence of congenital heart disease worldwide: A systematic review and meta-analysis. Journal of the American College of Cardiology.

[4] Bevilacqua F, Palatta S, Mirante N, Cuttini M, Seganti G, Dotta A, Piersigilli F (2013). Birth of a child with congenital heart disease: Emotional reactions of mothers and fathers according to time of diagnosis. The Journal of Maternal-Fetal & Neonatal Medicine.

[5] Albuquerque S, Fonseca A, Pereira M, Nazaré B, Canavarro M (2011). Estudos psicométricos da versão Portuguesa da Escala de Impacto Familiar (EIF). Laboratório de Psicologia.

[6] Fonseca A, Nazaré B, Canavarro MC (2012). Parental psychological distress and quality of life after a prenatal or postnatal diagnosis of congenital anomaly: A controlled comparison study with parents of healthy infants. Disability and Health Journal.

[7] Edraki M, Kamali M, Beheshtipour N, Amoosgar H, Zare N, Montaseri S (2014). The effect of educational program on the quality of life and self-efficacy of the mothers of the infants with congenital heart disease: A randomized controlled trial. IJCBNM.

[8] West CA, Besier T, Borth-Bruhns T, Goldbeck L (2009). Effectiveness of a family-oriented rehabilitation program on the quality of life of parents of chronically ill children. Klinische Padiatrie.

[9] Goldbeck L, Hölling I, Schlack R, West C, Besier T (2011). The impact of an inpatient family-oriented rehabilitation program on parent-reported psychological symptoms of chronically ill children. Klinische Padiatrie.

[10] Griffin T (2002). Supporting families of infants with congenital heart disease. Newborn and Infant Nursing Reviews.

[11] Awoyale T, Onajole AT, Ogunnowo BE, Adeyemo WL, Wanyonyi KL, Butali A (2016). Quality of life of family caregivers of children with orofacial clefts in Nigeria: A mixed-method study. Oral Diseases.

[12] Lazarus RS, Folkman S (1984). Stress, appraisal, and coping.

[13] Lukens, E. (2015). Psychoeducation - Social Work - Oxford Bibliographies.

[14] Arafa MA, Zaher SR, El-Dowaty AA, Moneeb DE (2008). Quality of life among parents of children with heart disease. Health and Quality of Life Outcomes.

[15] Landolt MA, Buechel EV, Latal B (2011). Predictors of parental quality of life after child open heart surgery: A 6-month prospective study. Journal of Pediatrics.

[16] Lawoko S, Soares JJF (2003). Quality of life among parents of children with congenital heart disease, parents of children with other diseases and parents of healthy children. Quality of Life Research.

[17] Solberg Ø, Grønning Dale MT, Holmstrøm H, Eskedal LT, Landolt MA, Vollrath ME (2011). Long-term symptoms of depression and anxiety in mothers of infants with congenital heart defects. Journal of Pediatric Psychology.

[18] Higgins J, Green S (2008). Cochrane handbook for systematic reviews of interventions.

[19] Cohen J (1988). Statistical power analysis for the behavioral sciences.

[20] Moher D, Liberati A, Tetzlaff J, Altman DG, Altman D, Antes G, Prisma Group (2009). Preferred reporting items for systematic reviews and meta-analyses: The PRISMA statement. PLoS Medicine.

[21] Hancock HS, Pituch K, Uzark K, Bhat P, Fifer C, Silveira M, Yu S, Welch S, Donohue J, Lowery R, Aiyagari R (2018). A randomised trial of early palliative care for maternal stress in infants prenatally diagnosed with single-ventricle heart disease. Cardiology in the Young.

[22] Van Der Mheen M, Van Beynum IM, Dulfer K, Van Der Ende J, Van Galen E, Duvekot J, Rots LE, Van Den Adel TP, Bogers AJ, McCusker CG, Casey FA (2018). The CHIP-Family study to improve the psychosocial wellbeing of young children with congenital heart disease and their families: Design of a randomized controlled trial. BMC Pediatrics.

[23] van der Mheen M, Meentken MG, van Beynum IM, Van Der Ende J, Van Galen E, Zirar A, Aendekerk EW, Van Den Adel TP, Bogers AJ, McCusker CG (2019). Hillegers MHCHIP-Family intervention to improve the psychosocial well-being of young children with congenital heart disease and their families: Results of a randomised controlled trial. Cardiology in the Young.

[24] Zhang QL, Xu N, Huang ST, Cao H, Chen Q (2021). WeChat-assisted pre-operative health education improves the quality of life of parents of children with ventricular septal defects: A prospective randomised controlled study. Journal of Paediatrics and Child Health.

[25] Zhang QL, Lei YQ, Liu JF, Cao H, Chen Q (2021). Using telemedicine to improve the quality of life of parents of infants with CHD surgery after discharge. International Journal for Quality in Health Care.

[26] Xie W, Liu J, Lei Y, Cao H, Chen Q (2021). Effects of WeChat follow-up management of infants who underwent ventricular septal defect repair on parents’ disease knowledge and quality of life: A prospective randomized controlled study. Journal of Cardiac Surgery.

[27] Leuthner SR, Bolger M, Frommelt M, Nelson R (2003). The impact of abnormal fetal echocardiography on expectant parents’ experience of pregnancy: A pilot study. Journal of Psychosomatic Obstetrics and Gynecology.

[28] Fonseca A, Nazaré B, Canavarro MC (2014). Parenting an infant with a congenital anomaly: An exploratory study on patterns of adjustment from diagnosis to six months post birth. Journal of Child Health Care.

[29] Fonseca A, Nazaré B, Canavarro MC (2014). The role of satisfaction with social support in perceived burden and stress of parents of six-month-old infants with a congenital anomaly: Actor and partner effects. Journal of Child Health Care.

[30] Vonneilich N, Lüdecke D, Kofahl C (2016). The impact of care on family and health-related quality of life of parents with chronically ill and disabled children. Disability and Rehabilitation.

[31] Yildiz A, Celebioglu A, Cardiologist P, Faculty M (2009). Distress levels in Turkish parents of children with congenital heart disease. The Australian Journal of Advanced Nursing.

