# Supplementary material for: Improving the quality of life of parents of patients with congenital abnormalities using psychoeducational interventions: a systematic review
Source: Qual Life Res. 2023 Jun 17;32(11):3027–37. doi: 10.1007/s11136-023-03452-8 (PMC10522751; doi:10.1007/s11136-023-03452-8)
Supplement: Supplementary file 1 — Supplementary file1 (PDF 2766 kb) [file 11136_2023_3452_MOESM1_ESM.pdf]

Attachment 1: Search strategy (14th May 2022)

P – Studies on parents of children with congenital abnormalities

I – Psychoeducational interventions

C – Standard care

O – Quality of life (QoL)

S – Quantitative comparative observational or experimental studies

How effective are psychoeducational interventions described for improving the QoL of parents of children with congenital malformations?

Sentinel articles used to test the query search:

M. Edraki, M. Kamali, N. Beheshtipour, H. Amoosgar, N. Zare, and S. Montaseri, "The effect of educational program on the quality of life and self-efficacy of the mothers of the infants with congenital heart disease: a randomized controlled trial," *IJCBNM*, vol. 2, no. 1, pp. 51–59, 2014.

L. Goldbeck, I. Holling, R. Schalack, C. West and T. Besier, "The impact of an inpatient family-oriented rehabilitation program on parent-reported psychological symptoms of chronically ill children," Klin. Padiatr., vol. 223, no. 2, pp. 78–84, 2011.

Pubmed

Components	***Query search***	***Results***
Parents	parents[MeSH Terms] OR parent* OR mothers[MeSH Terms] OR mother* OR maternal OR fathers[MeSH Terms] OR father* OR paternal OR family OR famil*	2,482,018
Congenital abnormalities<br><br>congenital heart defects	congenital abnormalities[MeSH Terms] OR (congenital AND abnormalit*) OR deformit* OR (congenital AND defect*) OR (birth AND defect*) OR (congenital AND disorder*) OR congenital heart defects[MeSH Terms] OR (congenital AND heart AND defect*) OR malformation of heart OR (heart AND abnormalit*) OR congenital heart disease* OR congenital heart condition* OR congenital heart disorder*	791,856<br><br>237,976<br><br>846,248
QoL	quality of life[MeSH Terms] OR quality of life OR life quality OR health related quality of life OR health-related quality of life OR health status[MeSH Terms] OR (health AND status) OR (health AND level*)	1,876,460
Education	health education[MeSH Terms] OR health education OR education OR counseling[MeSH Terms] OR counseling OR counselling OR psycho-education* OR psychoeducation* OR (educational AND program*) OR (educational AND intervention*) OR training	2,657,709
QUERY:<br>*Parents +*<br>*Congenital abnormalities (congenital heart defects)*<br>*+ QoL*<br>*+ Education*	(parents[MeSH Terms] OR parent* OR mothers[MeSH Terms] OR mother* OR maternal OR fathers[MeSH Terms] OR father* OR paternal OR family OR famil*) AND (congenital abnormalities[MeSH Terms] OR (congenital AND abnormalit*) OR deformit* OR (congenital AND defect*) OR (birth AND defect*) OR (congenital AND disorder*) OR congenital heart defects[MeSH Terms] OR (congenital AND heart AND defect*) OR malformation of heart OR (heart AND abnormalit*) OR congenital heart disease* OR congenital heart condition* OR congenital heart disorder*) AND (quality of life[MeSH Terms] OR quality of life OR life quality OR health related quality of life OR health-related quality of life OR health status[MeSH Terms] OR (health AND status) OR (health AND level*)) AND (health education[MeSH Terms] OR health education OR education OR counseling[MeSH Terms] OR counseling OR counselling OR psycho-education* OR psychoeducation* OR (educational AND program*) OR (educational AND intervention*) OR training)	**2,657**

SCOPUS – <u>article title, abstract, keywords</u>

Components	***Query search***	***Results***
Parents	parent* OR mother* OR maternal OR father* OR paternal OR family OR famil*	3,780,182
Congenital abnormalities<br><br>congenital heart defects	(congenital AND abnormalit*) OR deformit* OR (congenital AND defect*) OR (birth AND defect*) OR (congenital AND disorder*) OR (congenital AND heart AND defect*) OR "malformation of heart" OR (heart AND abnormalit*) OR "congenital heart disease*" OR "congenital heart condition*" OR "congenital heart disorder*"	444,513<br><br>179,487<br><br>530,652
QoL	"quality of life" OR "life quality" OR "health related quality of life" OR "health-related quality of life" OR (health AND status) OR (health AND level*)	1,882,794
Education	"health education" OR education OR counseling OR counselling OR psycho-education* OR psychoeducation* OR (educational AND program*) OR (educational AND intervention*) OR training	3,558,480
QUERY:<br>*Parents +*<br>*Congenital abnormalities (congenital heart defects)*<br>*+ QoL*<br>*+ Education*	(parent* OR mother* OR maternal OR father* OR paternal OR family OR famil*) AND ((congenital AND abnormalit*) OR deformit* OR (congenital AND defect*) OR (birth AND defect*) OR (congenital AND disorder*) OR (congenital AND heart AND defect*) OR "malformation of heart" OR (heart AND abnormalit*) OR "congenital heart disease*" OR "congenital heart condition*" OR "congenital heart disorder*") AND ("quality of life" OR "life quality" OR "health related quality of life" OR "health-related quality of life" OR (health AND status) OR (health AND level*)) AND ("health education" OR education OR counseling OR counselling OR psycho-education* OR psychoeducation* OR (educational AND program*) OR (educational AND intervention*) OR training)	**1,438**

***WEB OF SCIENCE* – <u>Topic (TS)</u>**

Components	***Query search***	***Results***
Parents	TS=(parent* OR mother* OR maternal OR father* OR paternal OR family OR famil*)	2,842,749
Congenital abnormalities<br><br>congenital heart defects	TS=((congenital AND abnormalit*) OR deformit* OR (congenital AND defect*) OR (birth AND defect*) OR (congenital AND disorder*)) OR TS=((congenital AND heart AND defect*) OR malformation of heart OR (heart AND abnormalit*) OR congenital heart disease* OR congenital heart condition* OR congenital heart disorder*)	173,244<br><br>93,063<br><br>238,023
QoL	TS=(quality of life OR life quality OR health related quality of life OR health-related quality of life OR (health AND status) OR (health AND level*))	1,359,324
Education	TS=(health education OR education OR counseling OR counselling OR psycho-education* OR psychoeducation* OR (educational AND program*) OR (educational AND intervention*) OR training)	2,383,675
QUERY:<br>*Parents +*<br>*Congenital abnormalities (congenital heart defects)*<br>*+ QoL*<br>*+ Education*	TS=(parent* OR mother* OR maternal OR father* OR paternal OR family OR famil*) AND (TS=((congenital AND abnormalit*) OR deformit* OR (congenital AND defect*) OR (birth AND defect*) OR (congenital AND disorder*)) OR TS=((congenital AND heart AND defect*) OR malformation of heart OR (heart AND abnormalit*) OR congenital heart disease* OR congenital heart condition* OR congenital heart disorder*)) AND (TS=(quality of life OR life quality OR health related quality of life OR health-related quality of life OR (health AND status) OR (health AND level*))) AND (TS=(health education OR education OR counseling OR counselling OR psycho-education* OR psychoeducation* OR (educational AND program*) OR (educational AND intervention*) OR training))	**577**

CENTRAL – title, abstract, keywords

Components	***Query search***	***Results***
Parents	[mh parents] OR parent* OR [mh mothers] OR mother* OR maternal OR [mh fathers] OR father* OR paternal OR family OR famil*	125,576
Congenital abnormalities<br><br>congenital heart defects	[mh “congenital abnormalities”] OR (congenital AND abnormalit*) OR deformit* OR (congenital AND defect*) OR (birth AND defect*) OR (congenital AND disorder*) OR [mh “congenital heart defects”] OR (congenital AND heart AND defect*) OR “malformation of heart” OR (heart AND abnormalit*) OR “congenital heart disease*” OR “congenital heart condition*” OR “congenital heart disorder*”	14,263<br><br>6,835<br><br>17,775
QoL	[mh “quality of life”] OR “quality of life” OR “life quality” OR “health related quality of life” OR “health-related quality of life” OR [mh “health status”] OR (health AND status) OR (health AND level*)	226,280
Education	[mh “health education”] OR “health education” OR education OR [mh counseling] OR counseling OR counselling OR psycho-education* OR psychoeducation* OR (educational AND program*) OR (educational AND intervention*) OR training	200,990
QUERY:<br>*Parents +*<br>*Congenital abnormalities (congenital heart defects)*<br>*+ QoL*<br>*+ Education*	([mh parents] OR parent* OR [mh mothers] OR mother* OR maternal OR [mh fathers] OR father* OR paternal OR family OR famil*) AND ([mh “congenital abnormalities”] OR (congenital AND abnormalit*) OR deformit* OR (congenital AND defect*) OR (birth AND defect*) OR (congenital AND disorder*) OR [mh “congenital heart defects”] OR (congenital AND heart AND defect*) OR “malformation of heart” OR (heart AND abnormalit*) OR “congenital heart disease*” OR “congenital heart condition*” OR “congenital heart disorder*”) AND ([mh “quality of life”] OR “quality of life” OR “life quality” OR “health related quality of life” OR “health-related quality of life” OR [mh “health status”] OR (health AND status) OR (health AND level*))<br>AND ([mh “health education”] OR “health education” OR education OR [mh counseling] OR counseling OR counselling OR psycho-education* OR psychoeducation* OR (educational AND program*) OR (educational AND intervention*) OR training)	**72**

PSYCINFO – <u>title, abstract and keywords</u>

Components	***Query search***	***Results***
Parents	MA parents OR parent* OR MA mothers OR mother* OR maternal OR MA fathers OR father* OR paternal OR family OR famil*	721,165
Congenital abnormalities<br><br>congenital heart defects	MA congenital abnormalities OR (congenital AND abnormalit*) OR deformit* OR (congenital AND defect*) OR (birth AND defect*) OR (congenital AND disorder*) OR MA congenital heart defects OR (congenital AND heart AND defect*) OR malformation of heart OR (heart AND abnormalit*) OR congenital heart disease* OR congenital heart condition* OR congenital heart disorder*	8,094<br><br>1,774<br><br>8,957
QoL	MA quality of life OR quality of life OR life quality OR health related quality of life OR health-related quality of life OR MA health status OR (health AND status) OR (health AND level*)	266,525
Education	MA health education OR health education OR education OR MA counseling OR counseling OR counselling OR psycho-education* OR psychoeducation* OR (educational AND program*) OR (educational AND intervention*) OR training	830,124
QUERY:<br>*Parents +*<br>*Congenital abnormalities*<br>*(congenital heart defects)*<br>*+ QoL*<br>*+ Education*	(MA parents OR parent* OR MA mothers OR mother* OR maternal OR MA fathers OR father* OR paternal OR family OR famil*) **AND** (MA congenital abnormalities OR (congenital AND abnormalit*) OR deformit* OR (congenital AND defect*) OR (birth AND defect*) OR (congenital AND disorder*) OR MA congenital heart defects OR (congenital AND heart AND defect*) OR malformation of heart OR (heart AND abnormalit*) OR congenital heart disease* OR congenital heart condition* OR congenital heart disorder*) **AND** (MA quality of life OR quality of life OR life quality OR health related quality of life OR health-related quality of life OR MA health status OR (health AND status) OR (health AND level*)) **AND** (MA health education OR health education OR education OR MA counseling OR counseling OR counselling OR psycho-education* OR psychoeducation* OR (educational AND program*) OR (educational AND intervention*) OR training)	**2,820**

We consulted the following **congresses/scientific meetings**:

1) *American Academy of Pediatrics National Conference & Exhibition* from responsibility of *American Academy of Pediatrics* (AAP);

2) *Congress of the European Academy of Paediatric Societies* organized by *European Academy of Paediatrics* (EAP), *European Society of Paediatric and Neonatal Intensive Care* (ESPNIC) and *European Society for Paediatric Research* (ESPR);

3) *Excellence in Pediatrics Conference* (EIP) organized by *Excellence in Pediatrics Institute*;

4) *Annual International Conference on Psychology* from responsibility of *Psychology Research Unit of the Athens Institute for Education and Research* (ATINER);

5) *WAIMH World Congress* from *World Association for Infant Mental Health (WAIMH)*.

Attachment 2: Eligibility checklist

Improve the quality of life of parents of children with congenital abnormalities using psychoeducational interventions – A systematic review

Eligibility checklist

Study ID: ____________________________________

Screened by: _________________________________

1. Study design

Is the study a *quantitative comparative observational or experimental study*?

❑ Yes ❑ No **(exclude)** ❑ Can't tell

2. Participants

Did the study include *parents of children with congenital abnormalities*?

❑ Yes ❑ No **(exclude)** ❑ Can't tell

3. Intervention *VS* Comparator

Did the study evaluate a *psychoeducational intervention VS standard care*?

❑ Yes ❑ No **(exclude)** ❑ Can't tell

4. Outcome

Did the study address the primary outcome *quality of life* of parents of children with congenital abnormalities?

❑ Yes ❑ No **(exclude)** ❑ Can't tell

Should this study be included in the review?

❑ **YES** ❑ **NO** ❑ Can't tell

Attachment 3: Description of psychoeducational interventions, QoL results observed, and effect sizes estimated in included studies.

Study	Description of interventions	QoL results – Mean(SD)	Size effect | Cohen's d[CI95%]
Edraki et *al.* (2014) (7)	**Educational program for mothers**<br>Mothers were divided into seven *4-subject groups* and received the educational program through *four 90-minute sessions in 4 weeks*. This program was presented through power point and included *information about the disease, types of the disease, causes, symptoms, diagnostic tests, treatment, its effect on the infant and the family, coping methods, taking care of such infants at home, nutrition, preventing infection, vaccination, and medication*. A booklet of the educational program was given to participants.	**SF-36 ***<br>*Study group*<br>**T0** - PCS 43.9(29.5); MCS 29.0(29.4)<br>**T1** - PCS 47.6(28.1); MCS 45.3(25.6)<br>**T2** - PCS 47.2(27.9); MCS 41.4(26.0)<br><br>*Control group*<br>**T0** - PCS 46.0(30.4); MCS 27.0(27.1)<br>**T1** - PCS 46.1(29.2); MCS 26.6(27.2)<br>**T2** - PCS 45.6(29.9); MCS 26.7(26.0)<br><br>**T0** - before the intervention<br>**T1** - immediately after the intervention<br>**T2** - 2 mo. after the intervention	**T1**<br>PCS 0.06[-0.46; 0.58]<br>MCS 0.71[0.17; 1.25]<br><br>**T2**<br>PCS 0.06[-0.46; 0.58]<br>MCS 0.57[0.03; 1.10]
Hancock *et al.* (2018) (21)	**Early pediatric palliative care**<br>The pediatric palliative care team (constituted by physician, nurse practitioner, nurse, and social worker with paediatric palliative care specialisation and training) performed the intervention when the neonates were admitted for planned surgery (following birth but before the first-stage palliative surgery). The *initial palliative care consultation* did not vary from any other palliative care consultation performed in the hospital, with a duration of 45–90 minutes. *Follow-up visits* varied from one to four ~30-minute visits per week.<br>The *structured pediatric palliative care intervention* specifically included evaluation of the following themes: *maternal understanding of their child's diagnosis and its broader impact on the child's and family's lives; concerns regarding their child's physical symptoms; social support systems and additional life stressors; expectations and hopes for their child's medical care; and fears surrounding their child's diagnosis and treatments*. Each palliative care intervention addressed these themes and included a particular focus on three important questions: "What is your understanding of your baby's diagnosis and how it might affect his/her and your family's lives?"; "What are you and your family hoping for?"; and "What are you most worried about?" The answers to these questions informed the paediatric palliative care team support and recommendations provided, frequency and duration of follow-up visits, and content of subsequent interactions.	**PedsQL FIM**<br>**T2**<br>*Study group*<br>Total score 60.0(13.9)<br>Parent HRQOL summary 57.2(16.7)<br>Family functioning summary 65.4(18.6)<br><br>*Control group*<br>Total score 60.2(25.0)<br>Parent HRQOL summary 60.5(24.6)<br>Family functioning summary 68.4(23.4)<br><br>**T2** - neonatal hospital discharge (or 30 days) following the 1st stage of palliative surgery	**PedsQL FIM**<br>**T2**<br>Total score 0.01[-0.63; 0,65]<br>Parent HRQOL summary 0.16[-0.48; 0.80]<br>Family functioning summary 0.14[-0.50; 0.78]

Study	Description of interventions	QoL results – Mean(SD)	Size effect | Cohen's d[CI95%]
van der Mheen *et al.* (2019) (22,23)	**CHIP-Family Intervention**<br>It is a *psychosocial intervention* that consists of a *parent module* and a *child module*. Parents and children *participate in a separate* but simultaneously given, *6-h group workshop*.<br>The **parent workshop** focuses on problem prevention therapy, *psychoeducation*, general parenting skills, skills specific to parenting a child with CHD, and medical issues. The lunch break offers families more opportunities to interact and share (similar) experiences. During the workshop, parents receive a manual which contains an overview of the topics that will be covered during the workshop and a home assignment on problem prevention therapy. Parents also receive handouts and a teacher information leaflet. Approximately 4 weeks after the workshop, parents receive an **individual follow-up booster session**. Questions or worries that may have come up after the workshop regarding their child with CHD or their family members are discussed. Also, aspects of the workshop which have been (most) helpful for parents and will be helpful in the future are reviewed. Moreover, the session focuses on the problem prevention home assignment and on how to promote the future use of problem prevention therapy.<br>The **child workshop** consists of psychological exercises based on the evidence-based cognitive behavioural therapy Fun FRIENDS protocol and sports exercises.	**SF-36**<br>*Study group*<br>**T1** - Mothers: PCS 51.5(8.5); MCS 48.6(9.7)<br>Fathers: PCS 54.2(5.4); MCS 53.9(4.3)<br>**T2** – Mothers: PCS 53.5(7.0); MCS 48.1(11.0)<br>Fathers: PCS 54.2(4.7); MCS 54.6(2.6)<br><br>*Control group*<br>**T1** - Mothers: PCS 52.3(6.5); MCS 49.6(9.4)<br>Fathers: PCS 52.4(6.0); MCS 52.0(6.5)<br>**T2** - Mothers: PCS 53.5(5.6); MCS 50.2(7.4)<br>Fathers: PCS 52.2(7.4); MCS 53.1(4.1)<br><br>**T1** - baseline (2 wk. before intervention)<br>**T2** - follow-up (6 mo. after T1)	**SF-36**<br>**T2**<br>**Mothers**<br>PCS 0.03[-0.41; 0.47]<br>MCS 0.22[-0.22; 0.66]<br><br>**Fathers**<br>PCS 0.32[-0.15; 0.79]<br>MCS 0.44[-0.03; 0.91]
Zhang *et al.* (2021) (24)	**WeChat-assisted pre-operative health education**<br>It included two parts: the education module and the question-and-answer module: (i) The **education module** included related knowledge on ventricular septal defect disease, pre-operative care, family care, feeding, and complication management. Parents could view the module and learn at any convenient time. (ii) **Question and answers module**: One medical staff member of the team was on duty every day and was online in the WeChat group at 18:00–22:00 hours to address parents' problems, provide reminders and supervise regular outpatient reviews and remind parents of the operation time. The medical staff also guided the family members in the WeChat group to communicate, discuss, and share the care experience and encourage each other actively.	**WHOQOL-Bref** (pre-operative)<br>*Study group*<br>Physiological 12.5(1.9)<br>Psychological 14.8(2.8)<br>Social 14.3(2.4)<br>Environment 13.5(2.2)<br><br>*Control group*<br>Physiological 9.7(1.3)<br>Psychological 10.2(1.5)<br>Social 10.6(1.2)<br>Environment 9.9(1.6)	**WHOQOL-Bref**<br>Physiological 1.72[1.17; 2.27]<br>Psychological 2.05[1.46; 2.63]<br>Social 1.95[1.37; 2.52]<br>Environment 1.87[1.30; 2.43]

Study	Description of interventions	QoL results – Mean(SD)	Size effect | Cohen's d[CI95%]
Zhang *et al.* (2021) (25)	**WeChat-assisted post-operative health education**<br>It included two parts, the education module and the question and answer module, described as follows: (i) the **education module** included related knowledge on CHD, post-operative care, family care, feeding and management of complications. Parents could view the module and learn at any time that was convenient for them. (ii) The **question and answer module** included one medical staff member of the team that was on duty every day and was online in the WeChat group from 18:00 to 21:00 to address parents' problems. The medical staff also guided the family members to communicate, discuss and share their care experiences and encourage each other actively.	**WHOQOL-Bref** (1 mo. after surgery)<br>*Study group*<br>Physiological 15.6(3.1)<br>Psychological 16.2(2.9)<br>Social 16.5(3.0)<br>Environment 15.8(2.8)<br><br>*Control group*<br>Physiological 10.8(3.3)<br>Psychological 10.2(3.5)<br>Social 9.7(3.6)<br>Environment 9.9(3.1)	**WHOQOL-Bref**<br>Physiological 0.75[0.44; 1.06]<br>Psychological 1.87[1.50; 2.23]<br>Social 2.05[1.67; 2.42]<br>Environment 2.00[1.63; 2.37]
Xie *et al.* (2021) (26)	**WeChat-assisted post-operative health education**<br>It included two modules: the **educational module** and the **question-and-answer module**. It was divided into two parts, namely, **knowledge education** and **psychological education**. The content mainly included knowledge of VSD, post-operative home care and feeding, management of complications, monitoring neurological and motor development, the family's potential influence on the disease, and managing psychosocial problems. In the form of pictures, text, or video, parents could watch and learn at any convenient time. For parents with pessimism, anxiety, or depression, we could provide psychological counseling and support through the WeChat platform. In the question and answer module, a medical officer on duty on the team went online on the WeChat group from 18:00 to 22:00 every day to explain problems to parents and to remind and supervise regular outpatient re-examinations. The medical staff also guided the parents to actively communicate, discuss and share post-operative rehabilitation experiences and encourage each other.	**PedsQL FIM** (3 mo. after surgery)<br>*Study group*<br>Total score 70.8(7.1)<br>Family functioning summary 70.6(13.1)<br><br>*Control group*<br>Total score 62.6(6.3)<br>Family functioning summary 63.0(15.7)	**PedsQL FIM**<br>Total score 1.22[0.74; 1.70]<br>Family functioning summary 0.53[0.08; 0.97]

ABBREVIATIONS: CHD: Congenital Heart Diseases; SF-36: SF-36 Health Survey; PedsQL FIM: Pediatric Quality of Life Inventory Family Impact Module; WHOQOL-Bref: World Health Organization Quality of Life Bref.

* Summary measures scores (PCS and MCS) are computed using the scoring algorithms described on pages 4:3-4 of the *SF-36 Physical and Mental Health Summary Scales: A User's Manual* (32)

Interpretation of effect size values (presented by Cohen in 1988): Values of 0.20, 0.50, and 0.80 for Cohen's d are commonly considered to be indicative of small, medium, and large effects.
